# Microbial carcinogenic toxins and dietary anti-cancer protectants

**DOI:** 10.1007/s00018-017-2487-z

**Published:** 2017-02-25

**Authors:** Trevor W. Stone, L. Gail Darlington

**Affiliations:** 1 College of Medical, Veterinary and Life Sciences, University of Glasgow, West Medical Building, Glasgow, G12 8QQ UK; 2 Department of General Internal Medicine, Ashtead Hospital, Ashtead, Surrey KT21 2SB UK

**Keywords:** Diet, Cancer, Microbiome, Serine proteases, Dependence receptors, DCC

## Abstract

Several toxins are known which account for the ability of some bacteria to initiate or promote carcinogenesis. These ideas are summarised and evidence is discussed for more specific mechanisms involving chymotrypsin and the bacterial chymotryptic enzyme subtilisin. Subtilisin and *Bacillus subtilis* are present in the gut and environment and both are used commercially in agriculture, livestock rearing and meat processing. The enzymes deplete cells of tumour suppressors such as deleted in colorectal cancer (DCC) and neogenin, so their potential presence in the food chain might represent an important link between diet and cancer. Over-eating increases secretion of chymotrypsin which is absorbed from the gut and could contribute to several forms of cancer linked to obesity. Inhibition of these serine proteases by Bowman–Birk inhibitors in fruit and vegetables could account for some of the protective effects of a plant-rich diet. These interactions represent previously unknown *non-genetic* mechanisms for the modification of tumour suppressor proteins and provide a plausible explanation contributing to both the pro-oncogenic effects of meat products and the protective activity of a plant-rich diet. The data suggest that changes to farming husbandry and food processing methods to remove these sources of extrinsic proteases might significantly reduce the incidence of several cancers.

## Introduction

The predominant view of cancer aetiology is that the root cause lies at the genomic level, perhaps facilitated by exposure to radiation or toxic chemicals in the environment or diet. However, there are few, if any, single genetic changes which can generate the full picture of increased cell proliferation, motility, migration, extracellular matrix metabolism and tissue penetration. Rather, it is more likely that these various facets of oncogenesis involve a series of changes in different metabolic pathways. This view has been proposed and argued in detail previously with the conclusion that five or six steps are required, probably in a specific sequence, for a cell to acquire an adequate spectrum of oncogenic properties to generate a malignant cancer [1, 2].

But how might five or six genetic changes to cellular pathways arise in a limited number of possible sequences such that cells do not trigger apoptosis and are not detected by immune surveillance systems? It seems improbable that such a cascade of intrinsically unlikely events could occur sufficiently frequently to account for the overall human cancer rate of 39.6% quoted by the US National Cancer Institute (http://www.cancer.gov/about-cancer/what-is-cancer/statistics). The statistics would become more realistic if one or more of the cellular changes needed for oncogenesis were present continually over an extended period of time either as a generalised shift in physiological conditions or as a maintained or intermittent exposure to an external factor in the biological background. Incidental and relatively transient events such as brief exposure to radiation or contact with a mutagen might then lead to a long-lasting aberration of cell physiology which, superimposed upon the distorted background, could lead to cancer. This concept is of fundamental importance in considering cancer prevention, as it implies that identifying and eliminating just one of the constant, background influences might prevent many cases of carcinogenesis.

Indeed, several recent authors have commented on the possible inter-relationships between external, non-genetic influences and cancer initiation. Dejea et al. [3] noted that “environmental factors clearly affect colorectal cancer incidence but the mechanisms through which these factors function are unknown”, while a second group pointed out that “the sources and consequences of non-genetic variability in metastatic progression are largely unknown” [4]. It has even been proposed that external influences may be more important than genetic abnormalities in the generation of some cancers [5].

Three areas of research are especially relevant to this problem as they focus on the interface between individuals and the environment in relation to oncogenesis. These areas include the roles of bacteria, of diet and of obesity. However, despite the intense interest in the influence of these factors on cancer, much of the evidence for them is epidemiological and correlative, with few convincing explanations of how any of these areas could induce cancer development. This review is an attempt to bring together recent data on the role of bacteria and diet at the cellular level, in an attempt to develop an over-arching concept which links these factors. Our conclusion not only provides a plausible and satisfying explanation of these links but also suggests a global strategy which might substantially lower the incidence of many cancers in a simple and cost-effective manner.

### The bacterial microbiome and cancer

#### General considerations

It has been estimated that over 15% of newly diagnosed cancers are attributable to a bacterial cause [6, 7], a concept that was greatly supported by the discovery that many gastric cancers can be traced to infection with *Helicobacter pylori* [8], while links between a single species of bacterium and other specific types of tumour have since been claimed in a variety of cases [9–11]. However, if there were more generalised mechanisms by which microorganisms could alter cell function indirectly towards an oncogenic state, generating a pool of cells sensitised to subsequent molecular damage, the number of tumours caused by such indirect actions of microbes could be very much higher.

Experimental studies have confirmed that it is possible to induce tumours using bacteria including the gastrointestinal (GI) microbiota which can be involved in oncogenesis indirectly by promoting a generalised inflammatory response and immune activation in tissues outside the GI tract. This latter concept is crucially important, emphasising that bacterial involvement in cancer initiation may not be limited to local tissues—those in which the density of microbes is at its highest—but can promote oncogenesis in distant tissues [12].

Inflammation, with or without the involvement of bacteria, contributes significantly to the initiation and development of cancers and plays a major part in the progression to gastric carcinoma [8]. TNF-α, in particular, has established roles in cancer progression in bowel, liver, breast and other sites in mice as well as mammary carcinomata in humans [13]. Activation of Toll-like receptors (TLRs) by bacterial lipopolysaccharides is the key to the initiation of cancer: chronic inflammation in TLR4-deficient mice fails to induce tumour formation, whereas receptor over-expression promotes oncogenesis [14].

**Fig. 1:**
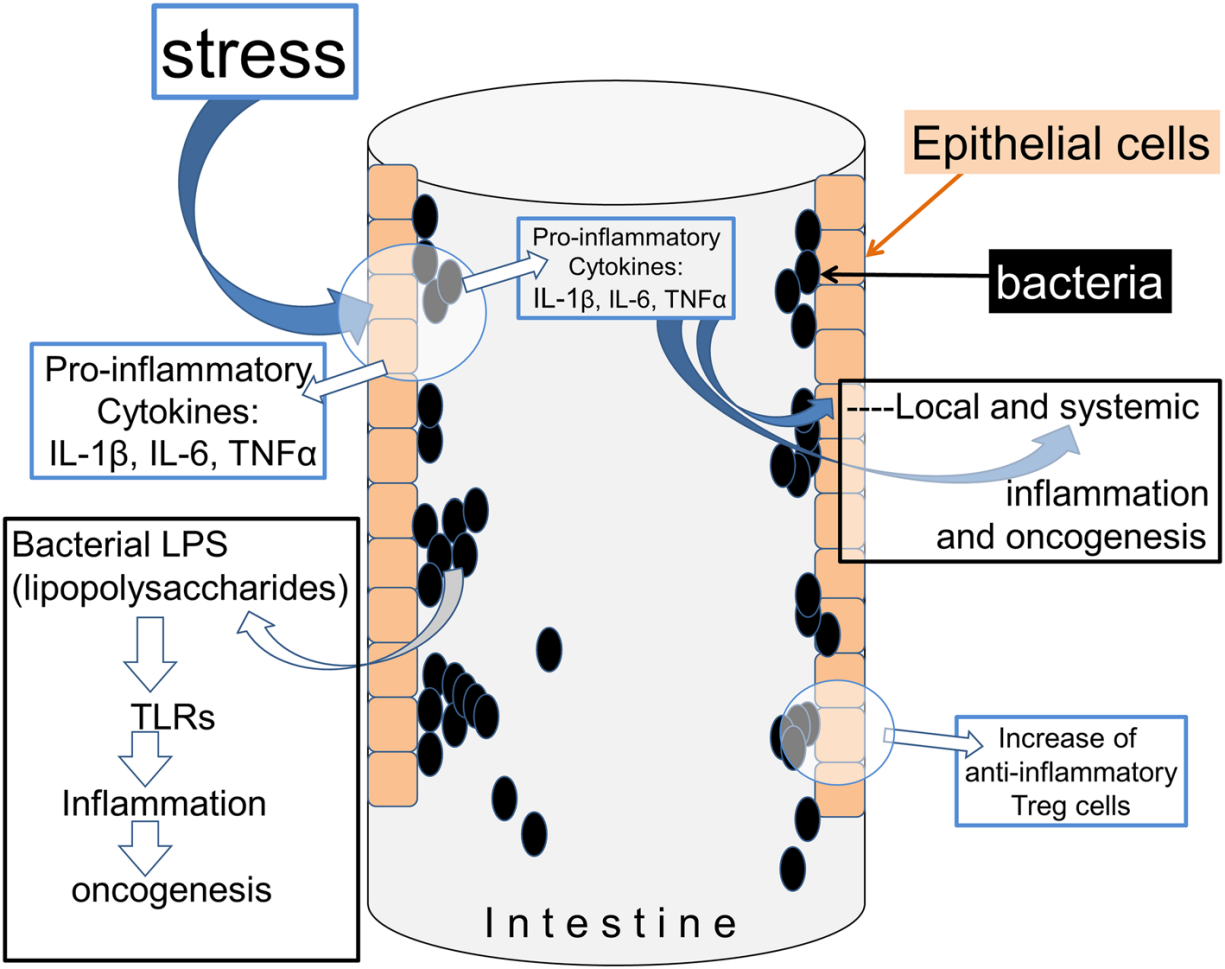
Stress can influence the interactions between bacteria and the host. The experience of stress can provoke the production of cytokines which act on bacteria, and the generation of cytokine-like molecules by bacteria which affect immune cell function in the host. The movement of T cells in particular between the chyme and peri-intestinal fluids can be affected, as well as the balance between different T cell subtypes and their respective generation of cytokines

In the intestinal mucosa, commensal bacteria play a role in maintaining the immune system generation of anti-inflammatory T-reg cells [14] (Fig. 1). Hence, interfering with the intestinal microbiota can potentially contribute to the development of a proinflammatory state which can, in turn, compromise the integrity of the mucosal barrier and lead to a more widespread and possibly systemic involvement [15]. Proinflammatory T cells, together with macrophages, are largely responsible for the production of cytokines such as interleukin-1β (IL-1β, IL-6, Tumour Necrosis Factor-α (TNF-α) and Transforming Growth Factor-β (TGF-β)), each of which has been linked with the promotion of oncogenic metabolism. TNF-α in particular has established roles in cancer progression in bowel, liver, breast and other sites in mice [16]. An association has also been made between levels of tissue TNF-α and human mammary carcinomata and together with an evidence that anti-inflammatory drugs reduce the incidence of breast cancer, this argues for a high relevance of inflammation in this disease [17].

**Fig. 2:**
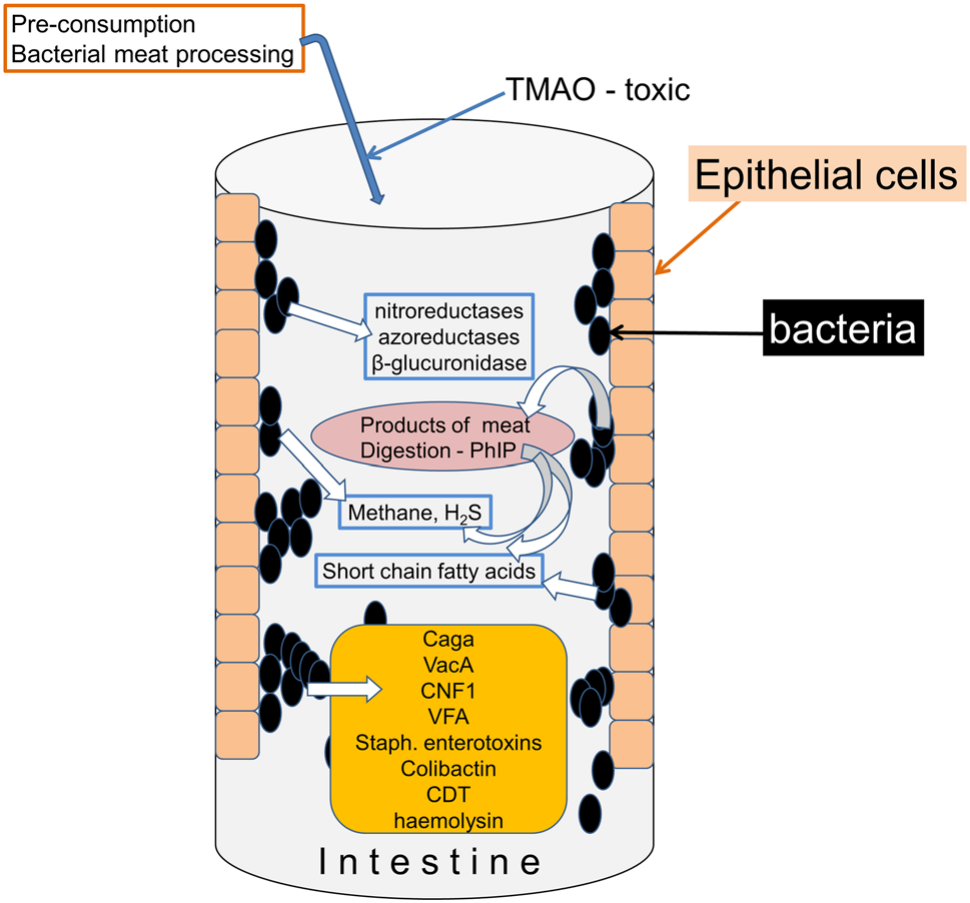
Known bacterial proteins and other toxins associated with cancer. Bacteria are known to produce several enzymes and smaller molecules, including inorganic compounds, which have linked with various forms of cancer

The gut microbiota may also be involved in cancer initiation produced by the impact of stress on the immune system (Fig. 2). Normally, exposure of animals to socially stressful situations results in increased blood levels of inflammatory mediators such as IL-1β, IL-6 and TNF-α and epinephrine [18], possibly as part of an acute-phase response. Similar stressful situations also altered the balance of microorganisms in the gut, and the immune activation produced by stress was prevented by removing the gut microbiota using high-dose antibiotics [19]. This not only highlights the sensitivity of the intestinal microbiota to environmental factors such as stress, but also indicates that the microbes are intimately involved in triggering the immune response to stress in the host mammal.

#### Microbial balance

The gastrointestinal (GI) microbiota account for around 90% of the cells present in the human body, but the composition of the microbiome varies significantly between individuals. There is good evidence that an important influence on oncogenesis is the balance between the several thousand species of bacteria which comprise the microbiome. Although the micro-flora and micro-fauna of an individual remain stable throughout adult life, they can be greatly altered by disease, dietary changes, stress or patterns of antibiotic use [20]. The ability of antibiotics to change the balance of organisms in the microbiome and the incidence of several cancers supports a role for microbiota in the development of those cancers [21].

Microorganisms exhibit numerous chemical methods by which a degree of ‘communication’ exists between different species and strains of bacteria, and between the microbial, mucosal and epithelial surfaces within the GI tract. The composition of the GI microbiota has been found to resemble that typical of individuals with cancers even before any malignancy has been detected, which may indicate that a bacterial factor could be among the earliest substances necessary for oncogenesis if—as predicted above—a continual baseline distortion of function is required upon which more transient perturbations are superimposed.

In particular, tumours are often associated with a lesser diversity of microbial species than normal, healthy tissue [22, 23] and probiotics can alter the overall mucosa-associated microbial profile. After an examination of the microbiota of patients with triple-negative breast cancer (TNBC), it was concluded that a ‘microbial signature’ was associated with the presence of TNBC which was not observed in normal tissue [24]. Thus, while individual bacterial species may have a dominant influence in some cancers, the overall microbial spectrum may modify that incidence and may, in some cases, mask a bacterial influence in some patients which might be a primary cause of disease in others. By generating a state of maintained low-level infection and inflammation, Enzler et al. [25] found that over 50% of the mice tested developed neoplasia but the formation of those tumours was prevented by treating the animals with a broad-spectrum antibiotic.

### Mechanisms of bacterial action

There are detailed reviews of the mechanisms by which prokaryotes might influence cancer development [26, 27]. A number of chemical substances consumed in the diet, generated within the intestine by endogenous enzymic activity or produced by the action of bacterial metabolism have been linked to the development of cancers, especially of the intestine (Fig. 2). They include overtly toxic and oncogenic compounds such as trimethyl-*N*-amine oxide (TMAO), produced by microbial digestion during preparation of processed meat products and linked with colorectal cancer [28], while 2-amino-1-methyl-6-phenylimidazo-[4,5-b]-pyridine (PhIP) is associated with the consumption of cooked red meat and prostatic cancer. Deoxycholic acid (DCA) is a secondary bile acid produced by many gut bacteria and has been linked with colon cancer promotion [27].

Even when bacteria are involved in their production, however, these compounds are likely to be produced by many of the species which inhabit the human GI tract. Equally, the effects of most of these compounds are non-specific in their actions, involving physico-chemical modifications of biologically significant host molecules with little selectivity in their target sites or mechanisms. Combatting insults with this level of randomness would be difficult to achieve.

#### Bacterial toxins

More amenable to pharmacological interference are biologically specific bacterial toxins (Fig. 2), although in many cases the detailed molecular mechanisms remain unclear. Several species of bacteria inhibit or degrade the tumour suppressor protein p53, including *Helicobacter pylori* in gastric epithelial cells [29]. Strains of *Staphylococcus* secrete toxic haemolysins and the Staphylococcal Enterotoxins promote proliferation of T cells, inducing normal cells to secrete factors that enhance proliferation of existing malignant T cells. *Salmonella typhi* can produce the protein toxin Virulence Factor A which alters cell proliferation directly or by increasing β-catenin expression [30]. The *Bacteroides fragilis* toxin (BFT) exists in an enterotoxin-producing form which induces inflammatory bowel disease and colorectal cancer [31].

Some of these more general toxins are potentially susceptible to agents which prevent their production, which block their binding sites on target molecules, which interrupt any host transduction pathways which are adversely hyperactivated, or which promote the activity of pathways which are down-regulated. Several of these toxins have been linked to the initiation or promotion of oncogenesis especially, but not exclusively, in the GI tract. More information is becoming available on their sites and mechanisms of action [32] and a few examples will be discussed next.

#### Vacuolating cytotoxin A (VacA)

*Helicobacter* species are among the bacteria most commonly associated with the development of cancer and are present in an estimated 50% of people worldwide. The stomach and duodenum are the regions most commonly involved, with gastritis and peptic ulcers progressing to various forms of gastric cancer. The main virulence factor produced by *Helicobacter* is Vacuolating Cytotoxin A (VacA). The binding of VacA to the gastric epithelium results in a marked proinflammatory response and increased cell proliferation, which is largely due to the formation of membrane porosities with secondary changes in macromolecular oligomerization [33]. It may be that the opening of membrane pores causes a disruption of diverse pathways depending on cell type and the local environmental conditions. Certainly, the production and secretion of VacA is partly dependent on local extracellular environment, being enhanced in high-salt (NaCl) concentrations but depressed by high levels of acidity [34].

#### Cytotoxin Associated Gene A (Caga)

Caga is the fourth most highly expressed protein in *H. pylori* and, accordingly, it has received the most intensive interest and investigation. Patients possessing *H. pylori* strains which express Caga are significantly more prone to develop gastric cancer than people with non-Caga carrying bacteria [35] with carcinogenesis being correlated with the amount of virulence gene expression [36]. Increases in the virulence of *H. pylori* induced by iron deficiency and high-salt consumption produce corresponding increases in cancer susceptibility.

Within the stomach, *H. pylori* cells become adherent to the gastric epithelial cells, where they avoid immunological detection and suppress host defence mechanisms locally and then proceed to invade the gastric mucosa. He et al. [32] identify the various virulence factors associated with these phases of disease, linking Caga and VacA expression with the later phases of transition from inflammatory status to oncogenesis. In addition, however, it was noted that polymorphisms in the host response genes for these factors were also important, including the E-cadherin receptors (CDH1) for Caga. Binding of Caga occurs at its Caga Multimerization (CM) motif and the existence of multiple polymorphisms of the CM probably accounts for the frequently observed variability in efficacy of the toxin. However, it is also recognised that there are strain variations in the *caga* gene promoter which affect the generation of some proinflammatory mediators such as IL-8 [37].

Caga is produced by the cag pathogenicity island [38] which is essential for gastric oncogenesis as it is responsible for inserting the Caga protein into host cells. The toxin inhibits PARtitioning-defective 1b (PAR1b), a serine–threonine kinase also known as Microtubule Affinity-Regulating Kinase-2 (MARK2), which plays a key role in determining cell polarity. The resulting disruption of tissue organisation compromises cell stability during proliferation and facilitates oncogenic transformation. These effects are compounded by the induction of proinflammatory processes within cells [39]. Another factor in the activity of Caga is the induced hypermethylation of tumour suppressor genes [40]. Caga increases the phosphorylation of protein kinase B (Akt), leading to activation of NFkB, up-regulation of DNA-(cytosine-5)-methyltransferase-1 (DNMT1) and tumour suppressor hypermethylation.

A number of other transduction pathways are susceptible to interference by Caga leading to disturbances of the epithelial mesenchymal transition (EMT), cell adhesion and migration. Among the pathways affected are Src homology 2-containing protein-tyrosine phosphatase-2 (SHP-2) and protein kinase C-related kinase-2 (PRK-2) which is inhibited by Caga [41]. A major role of the former enzyme is to regulate the activity of RhoGTPases which are the key players in the organisation and maintenance of the cytoskeleton. The promotion of EMT is accompanied by increased nuclear β-catenin and expression of the Snail1 and ZEB1 proteins. Although both caga and IL-1β can initiate EMT, only Caga increases cell invasiveness.

Sougleri et al. [42] observed the morphological and polarity changes characteristic of EMT in response to Caga. Using mutant gastric epithelial cells expressing variant forms of Caga with differing numbers of EPIYA (Glu-Pro-Ile-Tyr-Ala)-binding sites, they noted that phosphorylation of those sites mediates the interaction of Caga with SHP-2 noted above, leading to an elongated cell structure resembling that of the EMT and known as the ‘hummingbird’ phenotype. Variants of Caga with multiple EPIYA-binding sites are more motile and aggressive, showing a much greater propensity to induce gastric cancer. The mutant cells exhibited corresponding differences in the activation of matrix metalloproteinase-3 (MMP-3) with increased expression of the EMT markers Snail, ZEB1 and vimentin, as well as the stem cell marker CD44 [42].

TGF-β is an important regulator of tissue inflammation, but *H. pylori* Caga-positive cells inhibited TGF-β function via an interaction between Caga and Smad3 [43]. The depression in TGF-β anti-inflammatory activity resulted in increased secretion of IL-8 and other proinflammatory cytokines.

There are other actions of caga whose relevance to carcinogenesis remains uncertain. Caga produces increased proliferation, reduced apoptosis and increased secretion of extracellular matrix components from renal cells, leading to renal cancers [44] and it is required for the suppression by *H. pylori* of heat shock protein expression [45], an important aspect of the host cell response to infection and injury.

Importantly, the roles of *H.pylori* and Caga in disease are not confined to the stomach or intestine. The Caga protein is exported from the bacterial cells, as well as infected gastric epithelial cells, in the exosomes thus gaining wide distribution via the vascular circulation and potentially promoting carcinogenesis in a range of systemic tissues.

#### Cytolethal distending toxin (CDT)

Some Gram-negative bacteria including species of *Helicobacter*, especially those which target the liver such as *H. pullorum* and *H. hepaticus*, can generate another virulence factor cytolethal distending toxin (CDT) [46]. The B subunit (CdtB) of the toxin is the primary damaging element and induces NFkB translocation and activation with the expression of several proinflammatory markers and increased production of Th-17 cells. This proinflammatory spectrum probably underlies the progression to cancer formation which has been postulated for CDT [47]. Certainly, the chronic treatment of cells with CDT generates phenotypic changes characteristic of precancerous cells, with an increased frequency of mutation, chromosomal changes with enhanced genomic instability, p38MAPK activation and an increased ability to show anchorage-independent proliferation. There is also a marked increase in β-galactosidase activity (indicating senescence) and the production of proinflammatory cytokines including IL-6 and IL-8 [48]. The overall result is to extend cellular longevity and greatly enhance the probability of cancer formation [49].

There is also evidence for degradation by the DNAse activity of CDT as well as induced dysfunction of the DNA damage response [50]. This is known to occur in cells deficient in adenomatous polyposis coli (APC) or the p53 tumour suppressor [51]. The effect was claimed to be responsible for increasing the genomic instability seen in cells lacking APC or p53 and was probably responsible for the ability of the cells to show anchorage-independent growth. These factors led to the conclusion that, while there was little evidence that CDT could induce cancers directly, it would certainly promote the oncogenic consequences of APC or p53 mutations [51].

#### Cytotoxic necrotizing factor-1 (CNF1)

This toxin is produced primarily by the food-borne pathogen *Escherichia coli*, although some strains are normal, commensal organisms in the GI tract. At the N-terminus of the protein, there is a domain responsible for high-affinity binding to its target, with a nearby domain which promotes internalisation of the protein into cells [52]. The active site of CNF1 lies at the carboxyl terminus and promotes deamidation of glutamine in RhoGTPase enzymes. As a result, the cycle of GTP hydrolysis is blocked in the active state, leaving the target Rho enzymes permanently activated [53]. CNF1 can also facilitate the removal and degradation of Rho enzymes by increased ubiquitinylation and proteasomal metabolism via the Smurf1 pathway.

Several sites have been identified for the initial binding of CNF1. One of these is the Lutheran adhesion glycoprotein and Basal Cell Adhesion Molecule (Lu/BCAM) [54], while a second has been defined as the amino acid sequence 720–1014 which binds to the Laminin Precursor Protein p37LRP. A nearby high-affinity site is responsible for the attachment and adhesion of CNF1 to the cell membrane. The functional consequence of CNF1 binding is an increased cell proliferation and diminished the rate of senescence which can facilitate oncogenesis. CNF1 induces a proinflammatory phenotype in target cells with activation of NFkB, a release of proinflammatory cytokines from epithelial cells and increased cell migration [55].

CNF1 is one of the bacterial toxins whose carcinogenic properties are not confined to the GI tract. The toxin can be trafficked between cells via extracellular vesicles which are similar to, if not identical with, normal exosomes [56]. The toxin can therefore pass through the GI wall into other tissues, potentially transmitting its oncogenic activity to distant regions of the body.

#### Colibactin

Another toxic factor from *E. coli* is colibactin, a peptide-polyketide molecule secreted by group B2 *E. coli* [57, 58]. It is synthesised by a non-ribosomal peptide synthetase-polyketide synthase (pks) enzyme complex which is found in those strains of *E. coli* that are most often associated with GI tract tumours [58]. Colibactin produces significant DNA damage which, in addition to inflammatory activity and the induction of genomic instability, is thought to explain its association with cancer [59]. Colibactin possess an unique “warhead” responsible for interacting with DNA and producing cross-linking and strand breakages [60].

A related mechanism for cancer formation and stabilisation is the enhancement by colibactin of pathways which prolong tumour cell longevity. These include the generation of growth factors and senescence-related molecules such as microRNA-20-5p. The latter regulates Small Ubiquitin-like Modifier (SUMO) proteins with the accumulation of conjugates between SUMO and the tumour suppressor p53, leading to increased growth and proliferation.

### Novel bacterial targets: dependence receptors DCC and neogenin

Most bacterial products, as discussed above, affect general cellular pathways rather than those directly related to the cancerous properties of cells. Recent evidence has suggested that a more cancer-specific site—the dependence receptors—could be the target of an important group of bacterial and mammalian enzymes.

The ‘dependence receptors’ include three proteins which have been independently linked with cancer initiation and development: Deleted in Colorectal Cancer (DCC), neogenin and uncoordinated-5 (unc-5). The initial discovery that the *dcc* gene exhibited a loss of heterozygosity in many cases of colorectal cancer [61] was soon expanded with the realisation that similar deficits were seen in many other forms of cancer [62]. Abnormally low expression of DCC is indicative of poor patient prognosis [63, 64] and anti-sense DNA can increase rates of cell proliferation and migration [65, 66], whereas transfection with the ectopic DCC protein can have the opposite effect [66–68]. Increasing DCC expression even suppresses the pro-metastatic effects of depleting the tumour suppressor p53 [68].

**Fig. 3:**
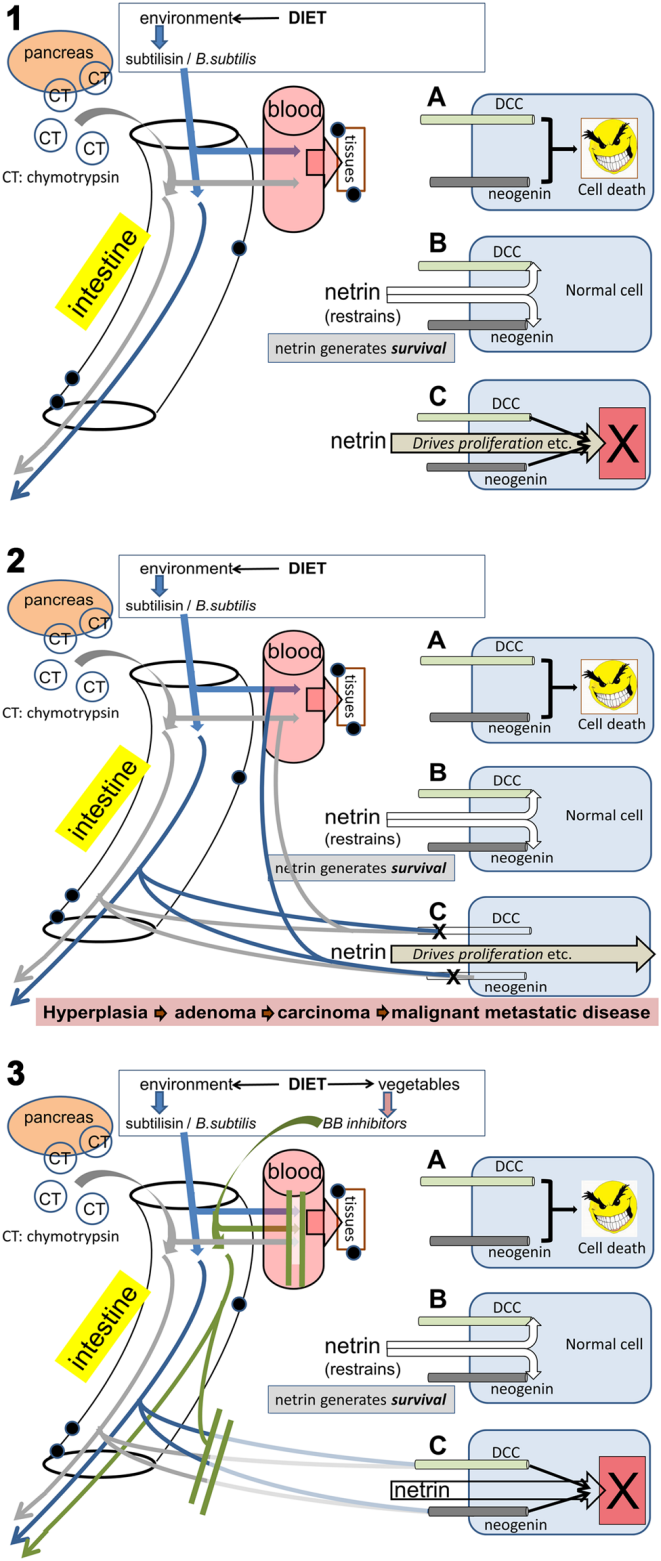
A schematic illustration of how serine proteases might link diet and obesity to cancer susceptibility. The *Panel 1 inset* (1 A) illustrates the presence of the dependence receptors (DRs) DCC and neogenin, which possess the intrinsic ability to initiate cell death. This activity is normally suppressed by the interaction of netrin with these receptors (1B) which restrains their apoptotic drive and allows cell survival. Conversely, the DRs inhibit the proliferative and migratory drive of netrin and if the DRs are blocked or deleted, netrin directly drives the cells to proliferate (1 C). The main part of *Panel 1* shows subtilisin (*blue arrows*) in the intestine, arising from commensal bacteria or dietary intake in meat products, with chymotrypsin (*grey arrows*) as a digestive enzyme potentially in elevated concentrations as a result of over-eating. Both are absorbed into the blood from where they can reach most organs and tissues. *Panel 2* illustrates the effect of subtilisin and chymotrypsin in the tissues, depleting DCC and neogenin (2 C) and permitting the netrin drive to over-proliferation and migration. This will promote the progression of cells to increasingly advanced stages of oncogenesis. The lower *panel 3* indicates the consumption of Bowman–Birk inhibitors in the diet and their absorption into the circulation and tissues where they can block the protease-mediated removal of the DRs, allowing their continued suppression of the netrin oncogenic drive (3 C)

DCC is a receptor for the extracellular secreted protein ligand netrin and functions as a brake on cellular apoptosis in the presence of netrin [69] (Fig. 3). If ambient levels of netrin fall, DCC is permitted to initiate apoptosis, thus ensuring that a damaged or isolated cell does not continue to exist for long even if it evades immune surveillance (which pro-cancerous cells appear to do at least partly by inducing indoleamine-2,3-dioxygenase (IDO) and its kynurenine metabolites) [70–72]. Conversely, a loss of DCC expression should prevent the initiation of apoptosis and allow cancerous cells to proliferate more readily. One factor in the suppression of cancer development, therefore, is the presence of a correctly functioning netrin-DCC axis and in the absence of DCC increased concentrations of netrin facilitate tumour formation [73], especially of the ovaries and breasts where it regulates mammary epithelial cell development. Netrin expression is also enhanced by NFkB, possibly contributing to the well-established association between chronic inflammation and cancer development.

#### Neogenin and Unc-5

A related protein, neogenin shows more than 50% sequence identity with DCC [73] and has broadly similar functions, with netrins and the family of Repulsive Guidance Molecules as extracellular ligands. There are important differences between DCC and neogenin, as in the early stages of embryogenesis when DCC expression is high and neogenin is low. Nevertheless, a reduction in neogenin expression can increase tumour formation by promoting proliferation, migration and invasion [74, 75], while over-expression can initiate apoptosis [76, 77]. Neogenin has been linked particularly with the development of mammary cancer [78, 79].

Finally, the Unc-5 receptor is also a tumour suppressor and its depletion from intestinal cells can promote cancerous behaviour [62, 80, 81].

In spite of the very significant role which these three dependence receptors play in the balance between apoptosis and proliferation, migration and invasion—a role which is clearly crucial in oncogenesis—functionally important gene mutations have been found relatively infrequently, raising the possibility that *non-genetic* abnormalities may underlie cellular dysfunction. One mechanism for that might be the recently described down-regulation of their expression by serine proteases, described next [82].

#### Serine proteases (SPs) deplete dependence receptors

Serine protease (SP) enzymes include the bacterial enzyme subtilisin and its mammalian equivalent chymotrypsin. They are able to modulate cellular communication by acting partly on proteasomal enzymes [83]. Recent work has found that both subtilisin and chymotrypsin are able to deplete the dependence receptors DCC and neogenin (and to a lesser degree Unc-5C) from mammalian cells [83–85], inducing cancerous properties such as increased migration [82]. This effect of SPs was selective, with several other proteins being resistant to the enzymes at concentrations 10- to 100-fold higher than those which affected DCC and neogenin. Subtilisin was active at nanomolar concentrations on SH-SY5Y human neuroblastoma cells and both subtilisin and chymotrypsin were active on SH-SY5Y cells, CaCo-2 human colorectal cancer cells, MCF-7 and MDA-MB-231 human mammary adenocarcinoma cells as well as on freshly isolated tissue, in which the levels of DCC are much higher than in cancer-derived cell lines. Since the cancer-derived cells expressed little DCC, the *dcc* gene was transfected into SH-SY5Y and MCF-7 cells. Subtilisin then decreased the expression of the ectopic DCC as well as the intrinsic neogenin in both cell lines. Both subtilisin and chymotrypsin significantly increased the migration of cells producing scratch closure in human MCF-7 breast cancer cells without any confounding changes in cell proliferation [82].

These results led to our formulating a new potential mechanism linking diet, bacteria, obesity and cancer (Fig, 3). Animals (including humans) are potentially exposed to subtilisin, firstly because it may be present in dietary products and secondly because *Bacillus subtilis* and related bacteria which secrete subtilisin are present in the intestine, the food chain and in cleaning materials (see below). Furthermore, chymotrypsin and trypsin levels are increased in individuals eating a high-meat diet and in obesity. In all these situations, the subtilisin or chymotrypsin may potentially deplete DCC and neogenin from cells of the GI tract, immune system cells in contact with the GI tract and in other tissues to which the enzymes have access after absorption. The following sections of this review will present more detailed information on these assertions.

#### Subtilisin in the environment and diet

Subtilisin is an alkaline serine protease and is classified as a chymotrypsin-like enzyme by virtue of its substrate specificity which overlaps that of chymotrypsin itself and is amenable to blockade by chymostatin. Conversely, many chymotrypsin-like enzymes are referred to collectively as subtilases and some have been linked with cancer initiation [86, 87].

Subtilisin is produced primarily by *Bacillus* species, although the term includes almost identical molecules such as subtilisin BPN from *B. amyloliquefaciens* and subtilisin Carlsberg from *B. licheniformis*. Closely related enzymes with similar substrate specificities are secreted by other bacteria including *Streptomyces* spp. and *Cryptosporidium* spp but are also produced by some fungi and yeasts such as *Aspergillus* species, *Cryphonectria parasitica, Trichoderma reesei*, which secretes a subtilisin-like protease in large quantities, and the ascomycete *Fusarium equiseti*.

*Bacillus subtilis* is a natural member of the microbiota present in the human GI tract, being isolated from ileal biopsies and faecal samples [88]. The bacteria and spores resist destruction in the stomach and intestine [89] and the surviving spores germinate in the intestinal lumen [90]. It is likely that *B. subtilis* remains a long-term inhabitant of the human intestine since it can exist aerobically (as in the atmosphere or soil) or anaerobically (as in the intestine) where it can survive and produce spores in the presence of nitrite or nitrate to furnish redox interacting ions as electron acceptors.

In the upper small intestine, the pH of many mammals including humans is around 6, but this rises as food proceeds through the lumen, reaching over pH 7 in the large bowel and sometimes exceeding pH 8 in patients with ulcerative colitis, a condition which can precede the development of colorectal cancer. Since subtilisin is an alkaline serine protease (optimal activity at pH around 8), it will function best in the large intestine and acidification of the bowel contents should inhibit the enzyme and its oncogenic properties on the dependence receptors [91]. A fibrous diet, which also tends to produce a more acid pH in the bowel, should contribute to the protective effects of dietary fibre and cereals against cancer [92] mediated by alkaline serine proteases.

Interestingly, high extracellular concentrations of salts increase the synthesis and secretion of several exoproteases from bacteria, including subtilisin and related enzymes [93], a phenomenon which may contribute to the high incidence of gastric cancers associated with the high-salt intake of the Japanese diet [94].

#### Industrial sources of subtilisin

As an enzyme with high stability at a wide range of temperature and acidity, subtilisin has found widespread commercial and industrial use. The bacterium *B. subtilis* shows a similar tolerance of the environment and of radiation than most common microorganisms. As a result, *B. subtilis* is found widely distributed in the environment and is found in high densities in soil.

In the food industry, subtilisin is used to tenderise meats to improve taste and texture and to facilitate packaging (approximately 1000 tonnes of the enzyme were used in Europe in 2007; [95]). It is also one of the several proteases used to digest those parts of animal carcasses that are unfit for human consumption and which may be used to feed animal livestock or to produce fertilisers [96], both uses of which might lead to subtilisin in the human food chain and, potentially, an increase in cancers in farm animals and humans.

In addition to these specialised industrial uses of subtilisin, the enzyme is a constituent of many detergent preparations used for cleaning in domestic and commercial establishments and for a variety of other industrial applications. The website of the US Household Products Database lists well over 100 items used in household cleaning which contain subtilisin (https://householdproducts.nlm.nih.gov/cgi-bin/household/brands?tbl=chem&id=1699). New variations in the structure of subtilisin are constantly being developed and patented for increased stability under atypical conditions of temperature and acidity in order to widen their range of industrial uses and markets.

#### Probiotics

The increasing use of probiotic preparations in agriculture, farming and medicine is being accelerated by the need to identify alternatives to conventional antibiotics to modify the GI microbiome towards a healthier mix and thus to promote animal growth. The *Bacillus* family includes several species found in probiotic preparations for use by humans and farm animals [97–99] including *B. subtilis, B. amyloliquefaciens and B. licheniformis*, all of which secrete subtilisin and which efficiently colonise the intestine with live bacteria, providing a valuable commercial advantage but with a potential oncogenic risk [89].

Whatever the gains in animal husbandry, there are risks associated with the handling of the ultimate product in the form of meat or carcasses. Marouani-Gadri et al. [100] examined the bacterial contamination of surfaces in a meat processing plant. Even after cleaning and disinfection, significant numbers of several bacterial species could be isolated, the most prominent being *Staphylococcus* and *Bacillus* species. Intensive rearing of animals in confined spaces is likely to exacerbate this problem. Beyond the food production industry, *B. subtilis* and related strains have been identified as contaminants in commercial fast food outlets [101].

#### Protease absorption and systemic cancers

While the GI tract would seem to be the tissue most likely to be traumatised by chymotrypsin or subtilisin in the diet or microbial secretions, it has long been recognised that large proteins including trypsin and chymotrypsin are absorbed from the GI tract [102–105] and reach the blood in physiologically relevant concentrations [105–107] (Fig. 3). Indeed, both proteins are far smaller (~27 kDa) than ferritin (~500 kDa) which is also absorbed [107]. Consequently, any increase in the GI levels of chymotrypsin or subtilisin, caused by obesity or dietary intake, respectively, will be reflected systemically, potentially generating an increased risk of cancer incidence in most internal organs.

#### Other chymotryptic proteases and subtilases

There are several other chymotryptic proteases in mammals in addition to those described above. A recent report indicates that neutrophils are required for the development of mammary tumours produced by bacteria [108]. The oral administration of *Helicobacter hepaticus* to mice usually resulted in the formation of mammary tumours but not if the blood was depleted of neutrophils by injections of the anti-Ly-6G antibody. One explanation of this could lie in the fact that inflammatory neutrophils, attracted to sites of tissue inflammation or infection and assisted by local mast cells, release large quantities of chymotryptic proteases, especially chymase and cathepsin G. These enzymes could then deplete mammary cells of DCC or neogenin [82], promoting the formation of tumours. This explanation fits perfectly with another recent observation that inflammation of the lungs can facilitate the formation of metastases which are dependent on serine proteases released from neutrophils [109].

A major argument favouring a role for serine proteases in cancer development is that they are specifically susceptible to inhibition by the Bowman–Birk inhibitors found in many dietary fruits and vegetables, the topic addressed below.

### Dietary plants and protection against cancer

After several decades of claims and counter-claims about the potential health benefits of foods or different plant species, some general principles are appearing for those plants that provide the strongest evidence. This review is focussed on general targets for tumour suppression, indicating specific compounds only where there is a strong bias of activity for a particular mechanism or if there is a target unique to that compound. Most of the following information relates to the anti-cancer activity of around ten major compounds (with their primary plant source): curcumin (turmeric), lycopene (tomato), resveratrol (red grapes, peanuts), genistein (soybean), sulforaphane (4-methylsulfinyl butyl isothiocyanate) and indole-3-carbinol (I3C and its dimerised metabolite, 3,3′-di-indolylmethane, DIM) (Brassica spp.), epigallocatechins (green tea), 6-gingerol (ginger), ellagic acid (pomegranate), β-carotene (carrots), diallyl sulphide and S-allyl cysteine (allium), allicin (garlic). In addition to these, the presence of Bowman–Birk inhibitors (see below) in a range of fruit and vegetables may be especially relevant in explaining the importance of a balanced meat and vegetable diet in which the plant component can block the effects of the serine proteases discussed above [82].

#### Cell cycle

Suppressing the cell cycle inevitably leads to the loss of tumour growth and, potentially, to the reduction of metastasis. Curcumin (diferuloylmethane) causes cycle arrest by up-regulating the tumour suppressors p53 and p21, a mechanism shared with compounds such as sulforaphane, other isothiocyanates, β-carotene, and N-methoxyindole-3-carbinol (NI3C) [110–112] leading to delays in the G0/G1 or G1/S transitions of the cycle.

Cessation of the cycle in the G2/M phases follows the activation of c-jun N-terminal kinase (JNK) and the p38 MAPK pathway. Sulforaphane normally halts cell progression in the G0/G1 and G2/M phases, but can stop proliferation in G1/G2 [113].

The cyclins and related proteins as well as the associated checkpoint kinases have been discussed in detail [114]. The cyclin-dependent kinases (cdk), especially cdk2 and cdk6, are particularly sensitive to inhibition by I3C and DIM and lead to cycle halting at the G2/M transition [115, 116]. However, the precise identity of the cyclins affected, the nature of their interactions and the results of interfering with them appear to vary substantially between cells of different cancer types. The cyclins are direct targets of several agents. Some flavonoids inhibit cdk4, cdk6 in addition to direct actions on cyclin D subtypes [117, 118], while others such as quercetin inhibit genes that induce mitosis, such as polo-like kinase-1 (PLK1) and cell division cycle protein 20 homolog (CDC20). Quercetin also has important actions on cell growth as well as proliferation [119]. The isothiocyanates, present in many species of *Brassica*, suppress expression of cdk1 and cdc25, partly by promoting their degradation, causing cycle stasis in G2/M. Resveratrol can arrest cells in the G1/S phase and induce apoptosis by interfering in cyclin–cdk interactions.

#### Cell death

Most of the pathways attacked by natural anti-cancer compounds result in a suppression of cell growth and proliferation, usually accompanied by the induction of apoptosis or autophagy. The Wnt/β-catenin pathway and associated proteins such as glycogen synthase kinase-3β (GSK-3β) and adenomatous polyposis coli (APC) are among the most frequently altered cellular molecules and they are influenced by many of the dietary compounds showing anti-cancer activity.

β-catenin promotes the expression of cell proliferation-related target genes, such as *c-myc* and cyclin D1 [120]. Curcumin suppresses the expression of p300 protein [121], an accessory molecule for the activation of oncogenic molecules by the Wnt/β-catenin pathway, thus inhibiting β-catenin-related transcription and depressing cell growth and proliferation. In the same work, *c-myc* was inhibited, contributing to cell cycle stasis in G2/M along with increased levels of apoptosis. Genistein up-regulates GSK-3β which, by retaining β-catenin in complexation with APC, prevents its nuclear translocation and gene activation [122]. DIM achieves the same result by enhancing phosphorylation of β-catenin, preventing its passage into the nucleus.

While the promotion of apoptosis is well established, there is less information on autophagy as a cancer-limiting mechanism. Certainly, many compounds of interest do target pathways implicated in autophagy, including the Ras-Raf/MAPK pathway, the Pi3KCI/Akt/mTOR sequence, FOXO1 signalling and p53. Antagonistic interactions also exist between pro- and anti-apoptotic pairs. The outstanding example of this is the Bcl-2 and Bcl-2 associated X protein (Bax) combination, the former apoptosis inhibitor being in competition with Bax and related proteins. Activity in the Bcl-2/Bax system is often initiated or enhanced under abnormal (stressful) conditions. Apoptosis is regulated by the actions of these proteins on the caspase cascade [123]. Bcl-2 and Bcl-extra large (Bcl-XL) can play key roles by mediating autophagic signalling pathways. Tumour suppressor p53 plays distinct roles in autophagy which depend on its subcellular localization. Nuclear p53 can promote autophagy by interacting with sestrins 1/2, but in the cytosolic compartment p53 inhibits autophagy by activating Bax.

As with many cellular functions, the existence of pathways critical to cell viability is accompanied by mechanisms to balance those functions, preventing cell damage or loss which might result from uncontrolled overactivity. Hence, the Inhibitors of Apoptosis Proteins (IAP) form several families of dietary targets whose suppression shifts the balance of activity in favour of apoptosis. Most of these proteins prevent caspase activation or promote their inactivation and degradation [123]. Sulforaphane down-regulates the anti-apoptotic gene Bcl-2 and the X-linked inhibitor of Apoptosis Protein (XIAP) [124].

The mammalian Target of Rapamycin (mTOR) is a negative regulator of autophagy in cancer cells and its phosphorylation mTOR is inhibited by curcumin and related compounds. The PI3KCI-Akt, Ras-Raf-1-MEK1/2-ERK1/2 signalling pathways present a route to mTORC1 which is, alternatively, susceptible to inhibition by the liver kinase B1 (LKB1)-AMPK complex [125]. The latter assembly down-regulated the drive to autophagy which is also influenced by the upstream interacting proteins PI3KCI-Akt. Both genistein and curcumin inhibit the Akt pathway, possibly as a result of inhibitory actions on src-family kinases, while I3C inactivates Akt via the inhibition of Pi3K expression and the loss of Akt phosphorylation [126]. Resting activity of PI3KCIAkt- mTORC1, or Akt alone, can normally be responsible for initiating autophagy, possibly making their activation by dietary compounds easier than inducing activity *ab initio*.

A major trigger for autophagy is the forkhead O transcription factor FOXO1. Chronically abnormal conditions in the cellular environment such as those likely to pertain during chronic inflammation induce FOXO1 to dissociate from its binding to sirtuin-2 (SIRT2). The subsequent acetylation state of FOXO1 is dependent on the balance of activity in epigenetic regulators such as histone acetylases and de-acetylases. The acetylated form of FOXO1 binds to the E1 accessory protein of Autophagy-related protein-7 (Atg7) to form the complex which regulates the initiation of autophagy [127].

The related target FOXO3 modulates the Akt/Wnt cascade discussed earlier. The binding of FOXO3 to the androgen receptor promoter is believed to be critical in prostate cancer and that binding is prevented by DIM. Other compounds affecting Akt, noted above, will also modify activity in the Akt/FOXO3a/GSK-3β/AR pathway [128]. The ellagitannin group of compounds shows beneficial effects against colon carcinogenesis by inhibiting Wnt signalling and by suppressing the PI3K/Akt pathway.

Cell demise, producing a loss of cancerous cells, can also be orchestrated by receptors responding to external ligands such as TNF-α. In most cases, cell death is mediated by the caspase cascades. Benzyl isothiocyanate and sulforaphane are examples of *Brassica* compounds able to promote this pathway to cancer cell death production [129].

#### Inflammation

Chronic inflammation is a major risk factor for the initiation of cancer. The reduced activation of MAPK by many dietary compounds noted above generates a secondary loss in expression of inflammation-related proteins such as cyclo-oxygenase-2 (COX-2) and interleukin-6 (IL-6), both of which can affect the production of TNF-α [130]. Many members of the flavonoid class, especially the flavonols, can decrease the expression of proinflammatory mediators such as IL-1α, IL-4, inducible nitric oxide synthase (iNOS) and TNFα directly [118].

The inflammation driver Nuclear Factor κ-light-chain-enhancer of activated B cells (NF-κB) is frequently activated either directly or by inhibition of its inhibitory component (inhibitor of kB kinase, IKK). The isoflavone genistein inhibits Notch signalling which results in the suppression of NFkB activity [131]. Curcumin and many flavonoids in food also inhibit NFkB and its downstream targets including cyclin D1, COX-2, MMP-9 and the Bcl-2 / Bcl-xL pathway, by down-regulating the expression of IKK. I3C and DIM also inhibit NFkB binding to DNA [118, 132]. Resveratrol prevents the activation of NF-kB and several inflammation-related genes with particularly marked suppression of iNOS [133]. The action of compounds on these pathways includes an inhibition of activation in response to external stimuli such as lipopolysaccharides (LPS) from bacterial infections. Flavonoids in fruits and vegetables are good regulators of NF-B expression. Their anti-inflammatory actions are enhanced by inhibiting ornithine decarboxylase and COX-2 activity as well as matrix metalloprotease expression [118].

#### Cell migration

An important class of enzymes intimately involved in cell migration is that of protein kinases. For abnormally active, cancerous cells, these provide a plethora of targets for plant-based compounds. Curcumin, I3C and DIM inhibit p38 MAPK and reduce the activity of several of the MAPK kinase kinase (MEKK) enzymes and JNK proteins [134]. Resveratrol also induces apoptosis by activation of MAPK, causing caspase activation and cell death [115, 130].

A number of anti-tumour compounds affect the endothelial mesenchymal transition (EMT), resulting in the suppression of cell migration and metastasis formation. Sulforaphane increases the epithelial expression of E-cadherin and so stabilises cells in their resting state, inhibiting metastasis. This is accompanied by a reduced expression of the Zinc finger E-box-binding homeobox 1 (Zeb1) which is often used as a characteristic marker of the early stages of EMT. Sulforaphane inhibits migration and invasion partly by activating ERK1/2 and downstream signalling. This leads to up-regulation of E-cadherin, the stabilisation of cell state and the suppression of EMT. Suppression of activity in the Wnt / GSK3β / β-catenin pathway (above) will contribute to the prevention or interruption of EMT and of cell migration.

An important aspect of tumour expansion and metastasis formation is the breaking of barriers to permit the ingress of invading cells. Among the most commonly targeted proteins in this respect are the matrix metalloproteases (MMPs), whose expression can be promoted by a variety of mechanisms including the increase of Notch signalling, for example, by sulforaphane which promotes MMP-9 expression.

Combining several compounds from these various groups, in a well-balanced diet, should bring substantial improvements to their efficacy as anti-cancer agents. Thus, combinations of epigallocatechin-3-gallate and sulforaphane (*Brassica* spp.) produced markedly greater improvement in the inhibition of breast cancer cell growth and progression of the disease in vivo than either compound alone [135, 136].

#### Aryl hydrocarbon receptor (AHR)

A different series of molecular targets for plant compounds are those associated with the metabolism of key regulators of cell function. Several dietary compounds can induce or enhance the activity of enzymes responsible for destroying foreign and toxic compounds, which would include some of the bacterial products and cooking-related toxins discussed earlier in this review. An example is the effect of sulforaphane to inhibit cytochrome P450 Phase I enzymes which normally catabolise heterocyclic amines produced by cooking into carcinogenic derivatives that interact with nucleic acids and disrupt gene transcription [137, 138]. The same compounds induce the expression of phase II metabolic enzymes which complex with potentially toxic compounds, especially those with marked pro-oxidant and mutagenic properties, to catabolise them for excretion.

One receptor-based mechanism is proving to be a potentially significant target of dietary compounds. Although long recognised as a xenobiotic sensing receptor, the Aryl Hydrocarbon Receptor (AHR) is now known to inhibit tumour development, partly because of its regulation of innate immune surveillance and response mechanisms. This activity is either constitutive or maintained by local levels of endogenous agonists such as kynurenine, since deletion of AHRs increases the entry of breast cancer cells into cycle stasis [139]. The dietary indole derivatives IC3 and DIM can activate the receptor to produce both a direct inhibition of cell proliferation and an inhibition of oestrogen-dependent tumorigenesis [140]; its possible activation by other dietary compounds is under intense investigation.

#### Serine protease inhibitors: the Bowman–Birk Factor

In considering the role of diet in cancer, meat intake represents only one part of the problem. The other is whether the anti-cancer effects of a plant-rich diet [141–143] can be explained by the actions of serine proteases (Fig. 3). Having discussed the possibility that serine proteases may contribute to carcinogenesis by depleting cells of DCC and neogenin, it is highly relevant that plants contain several serine protease inhibitors, the largest group of which is that of Bowman–Birk inhibitors (BBIs) [144–147]. BBIs are primarily inhibitors of chymotrypsin, although many have a second, independent catalytic site which inhibits trypsin. Members of this family occur in a wide range of plants including the widely studied inhibitor from soybean (*Glycine max*) as well as lentils, pulses, wheat, potatoes and many other edible plants [145]. There are numerous studies of the health and anti-cancer activity of BBIs [148], with comprehensive reviews of their activities and health benefits including anti-inflammatory and anti-cancer properties [144, 145].

Soybeans are not only water-soluble, but can also resist boiling for limited periods, with high stability over a range of temperatures and acidity in addition to their resistance to metabolism in the GI tract. The reports of serine protease depletion of DCC included the observation that the soybean BBI blocked the ability of chymotrypsin to down-regulate DCC and also blocked the increased migration of MCF-7 human breast adenocarcinoma cells produced by chymotrypsin [82]. This strongly supports the serine protease hypothesis and the anti-cancer protective effects of a plant-rich diet.

Despite some negative reports [149, 150], studies show consistently that some types of meat promote colon carcinogenesis in treated animals [151–157]. Similarly in human studies, there is a demonstrably higher risk of cancer, especially colorectal cancers, following regular and frequent meat consumption [158–167]. In one large-scale study, a vegetarian diet reduced the risk of colorectal cancer by 19% − 29% compared with non-vegetarians, while fish-eaters showed a 43% lower risk compared with the non-vegetarians [141]. These comparisons were reflected in other large cohorts which identified a significant link between colorectal cancer and the consumption of processed meat [158, 160, 163].

Overall, it seems clear that regular consumption of meat, especially of processed meat products, is probably linked to several forms of cancer and there are reasons to believe that serine proteases might be involved. Conversely, the consumption of a plant-based diet, rich in the protease inhibitory Bowman–Birk compounds, might protect against cancer by reducing the contribution of those serine proteases to the cancer risk (Fig. 3).

### Summary and the potential for prevention

This review has summarised some of the evidence that bacteria and their products may be involved in the association between a meat-based diet and oncogenesis, emphasising the selective effect of two serine proteases in depleting cells of the tumour suppressors DCC and neogenin, thus potentially promoting oncogenesis and metastasis. The bacterial protease subtilisin and *B. subtilis* which secretes it, may be employed in farm animal husbandry via probiotic administration, meat processing and some cleaning products. Their possible presence in the food chain and environment may predispose some individuals to develop a range of cancers. Mammalian chymotrypsin levels in the intestine and bloodstream are increased in obese individuals and those consuming a high-meat intake, possibly accounting for the link between obesity and cancer. The absorption of both proteases from the intestine may promote cancer in many internal organs as well as the GI tract. Perhaps most importantly, this mechanism may be part of the continual assault on cells from background, environmental sources, which primes some cells to the damage inflicted by more transient insults such as a toxin or radiation or which exacerbates the oncogenic potential of any genetic abnormality. The consumption of many fruits and vegetables will provide BBIs which inhibit serine proteases and may explain the protective effects of a plant-rich diet.

These concepts require extensive validation and expansion but, if they are correct, the implications for cancer prevention might be substantial. At the more difficult level of selectively removing specific bacterial sources of serine proteases, tools to kill the organisms responsible, such as species-specific siRNA or anti-sense oligonucleotides might be feasible. Certainly similar approaches to block the synthesis of subtilisin should achieve a similar objective.

If the importance of subtilisin and related serine proteases on DCC removal and cancer promotion is substantiated, population exposure to serine proteases could be readily produced by modifying agricultural and food processing methods, and increasing the intake of purified plant-derived enzyme inhibitors such as the Bowman–Birk families. Even the simple expedients of more thorough rinsing of kitchen and dining implements to remove residual films of bacteria, rinsing of clothing washed in ‘biological’ detergents containing subtilisin-like enzymes and the rigorous scrubbing of soil from un-peeled root vegetables could reduce the levels of domestic contamination by bacteria and their serine proteases. Together, such considerations might yield very cost-effective reductions in the global burden of many cancers.
